# Supplementary material for: Selection, Characterization, and Optimization of DNA Aptamers against Challenging Marine Biotoxin Gymnodimine-A for Biosensing Application
Source: Toxins (Basel). 2022 Mar 5;14(3):195. doi: 10.3390/toxins14030195 (PMC8949142; doi:10.3390/toxins14030195)
Supplement: Supplementary file 1 [file toxins-14-00195-s001.zip › toxins-1616959-supplementary.pdf]

toxins

MDPI

Supplementary Materials

Selection, Characterization, and Optimization of DNA Aptamers against Challenging Marine Biotoxin Gymnodimine-A for Biosensing Application

Xiaojuan Zhang [1,2,†], Yun Gao [1,†], Bowen Deng [1,†], Bo Hu [3], Luming Zhao [1], Han Guo [1], Chengfang Yang [1], Zhenxia Ma [1], Mingjuan Sun [1], Binghua Jiao [1,*] and Lianghua Wang [1,*]

[1] Department of Biochemistry and Molecular Biology, College of Basic Medical Sciences, Navy Medical University, Shanghai 200433, China; emilyzhangxj@126.com (X.Z.); gaoyun2014@sohu.com (Y.G.); dengbwgsy9373@gmail.com (B.D.); zlm19960726@163.com (L.Z.); ggatecnu@163.com (H.G.); nicole20220104@126.com (C.Y.); mazhenxia0515@sina.com (Z.M.); sunmj@smmu.edu.cn (M.S.)
[2] College of Medicine, Shaoxing University, 900th Chengnan Avenue, Shaoxing 312000, China
[3] Department of Marine Biomedicine and Polar Medicine, Naval Medical Center of PLA, Navy Medical University, Shanghai 200433, China; hb8601@163.com
* Correspondence: bhjiao@smmu.edu.cn (B.J.); lhwang@smmu.edu.cn (L.W.)
† These authors contributed equally to this work.

Table S1. Sequences of DNA oligonucleotides.

Name of ssDNA	Sequence （5′-3′）
F1	GGGAGGACGAAGCGGAAC
R1	TGTCGGGCGTGTCTTCTG
LR1	A_{20}-Spacer18-TGTCGGGCGTGTCTTCTG
Capture oligo	Biotin-GTC-Spacer18-GATCGAGCCTCA
Bank1	GGGAGGACGAAGCGGAAC-N10-TGAGGCTCGATC-N30-CAGAAGACACGCCCGACA
Random Library	GAGCCTTAGCAGCACTTCACACGATCCACCCTTAAGGCCAACCTTAACACGACCTCAAACACTGCGTAATGACTGTAGTGATGCCTTA

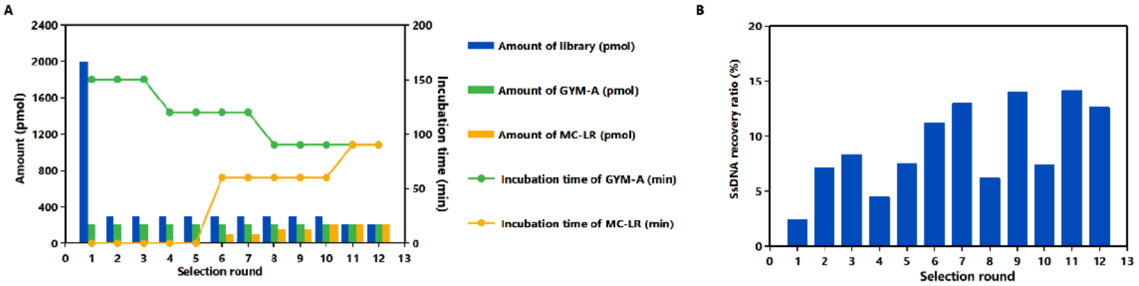

Figure S1. SELEX conditions for isolating aptamers toward GYM-A (A) and recovery ratio of ssDNA in capture-SELEX (B).

Table S2. SELEX conditions for isolating aptamers toward GYM-A.

Round	Amount of library (pmol)	Amount of GYM-A (pmol)	Amount of MC-LR (pmol)	Incubation time of GYM-A (min)	Incubation time of MC-LR (min)
1	2000	200	0	150	0
2	300	200	0	150	0
3	300	200	0	150	0
4	300	200	0	120	0
5	300	200	0	120	0
6	300	200	100	120	60
7	300	200	100	120	60
8	300	200	150	90	60
9	300	200	150	90	60
10	300	200	200	90	60
11	200	200	200	90	90
12	200	200	200	90	90

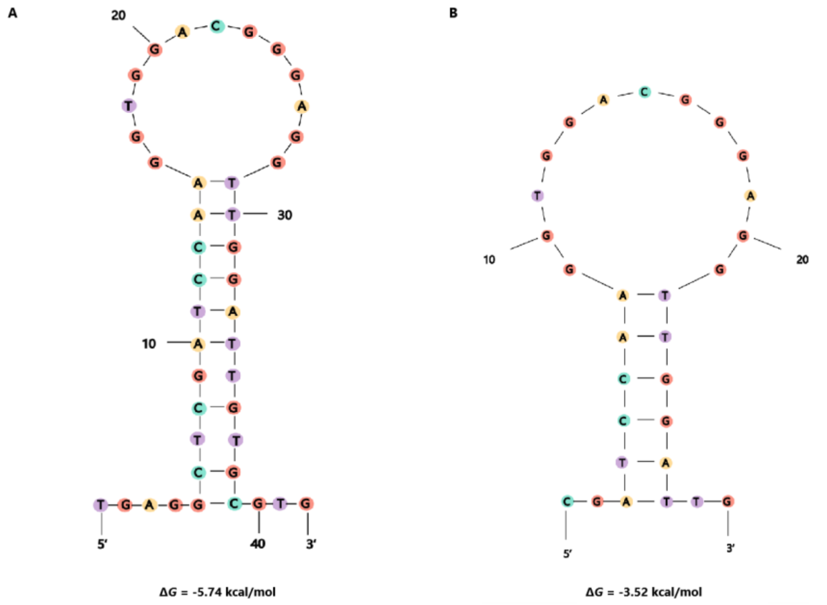

Figure S2. Secondary structure prediction of truncated aptamers of G48nors (A) and G48norsj (B) with their respective lowest Gibbs free energy value by mfold program. The folding temperature is 25 °C, and the concentrations of Na^+ and Mg^{2+} were 100 mM and 2 mM, respectively.

Table S3. Quadruplex forming G-Rich Sequences (QGRS) sequences found (overlaps included) in aptamer G48nop.

Position	Length	QGRS (5′-3′)	G-Score
14	21	GGCTCGATCCAAGGTGGACGG	12
14	22	GGCTCGATCCAAGGTGGACGGG	12
14	25	GGCTCGATCCAAGGTGGACGGGAGG	12
14	25	GGCTCGATCCAAGGTGGACGGGAGG	13
14	25	GGCTCGATCCAAGGTGGACGGGAGG	12
14	25	GGCTCGATCCAAGGTGGACGGGAGG	10
14	25	GGCTCGATCCAAGGTGGACGGGAGG	9
14	29	GGCTCGATCCAAGGTGGACGGGAGGTTGG	12
14	29	GGCTCGATCCAAGGTGGACGGGAGGTTGG	16
14	29	GGCTCGATCCAAGGTGGACGGGAGGTTGG	16
14	29	GGCTCGATCCAAGGTGGACGGGAGGTTGG	13
14	29	GGCTCGATCCAAGGTGGACGGGAGGTTGG	10
14	29	GGCTCGATCCAAGGTGGACGGGAGGTTGG	11
14	29	GGCTCGATCCAAGGTGGACGGGAGGTTGG	10
14	29	GGCTCGATCCAAGGTGGACGGGAGGTTGG	6
14	29	GGCTCGATCCAAGGTGGACGGGAGGTTGG	4
26	13	GGTGGACGGGAGG	20
26	13	GGTGGACGGGAGG	19
26	17	GGTGGACGGGAGGTTGG	16
26	17	GGTGGACGGGAGGTTGG	17
26	17	GGTGGACGGGAGGTTGG	16
26	17	GGTGGACGGGAGGTTGG	18
26	17	GGTGGACGGGAGGTTGG	16
29	14	GGACGGGAGGTTGG	21
29	14	GGACGGGAGGTTGG	19

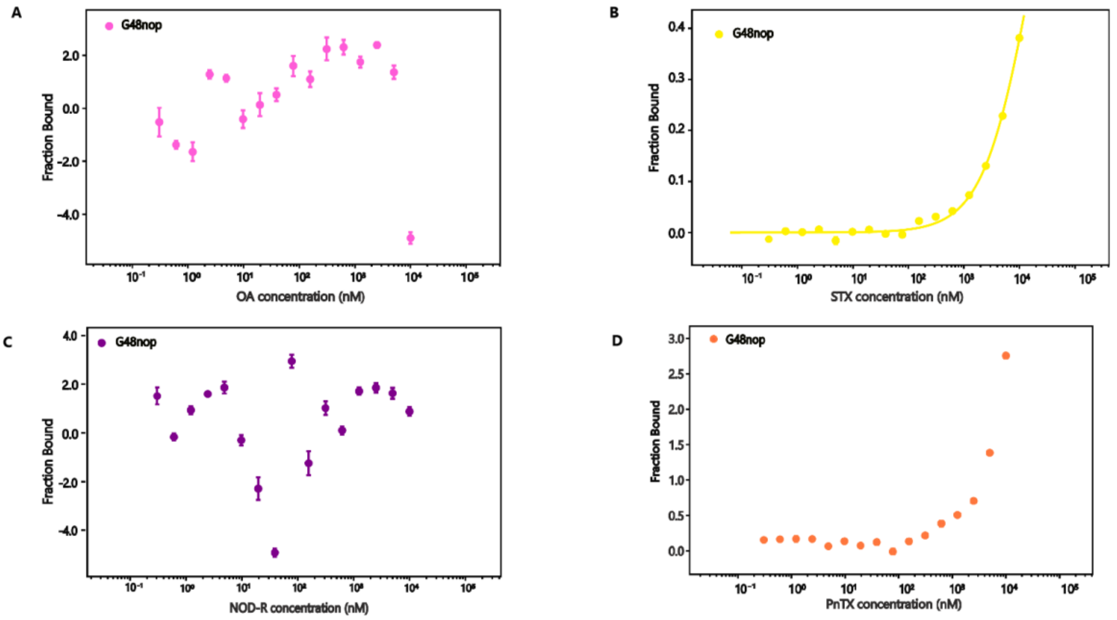

Figure S3. MST assays of aptamer G48nop for (A) OA, (B) STX, (C) NOD-R, and (D) PnTX. The K_D value of STX is 16,312.00 ± 3475.10 nM. G48nop shows no binding affinity for OA, NOD-R, and PnTX.

Table S4. Affinity constants (K_D) of aptamer G48nop for typical marine biotoxins toxins measured by MST assays.

Name of toxins	K_D (nM)
GYM-A	34.50 ± 1.72
OA	NB
STX	16,312.00 ± 3475.10
NOD-R	NB
PnTX	NB

NB stands for no binding.

Table S5. Measurement of inter and intra assay precision by aptasensor.

GYM-A concentration (nM)	Intra assay RSD (%) (n=3)	Inter assay RSD (%) (n=3)
55	1.12	4.57
109	0.80	1.35
219	0.49	3.69
438	0.49	2.18
875	3.14	2.32
1750	3.10	0.37
3500	2.59	1.39
7000	2.97	1.38
14,000	2.82	0.34

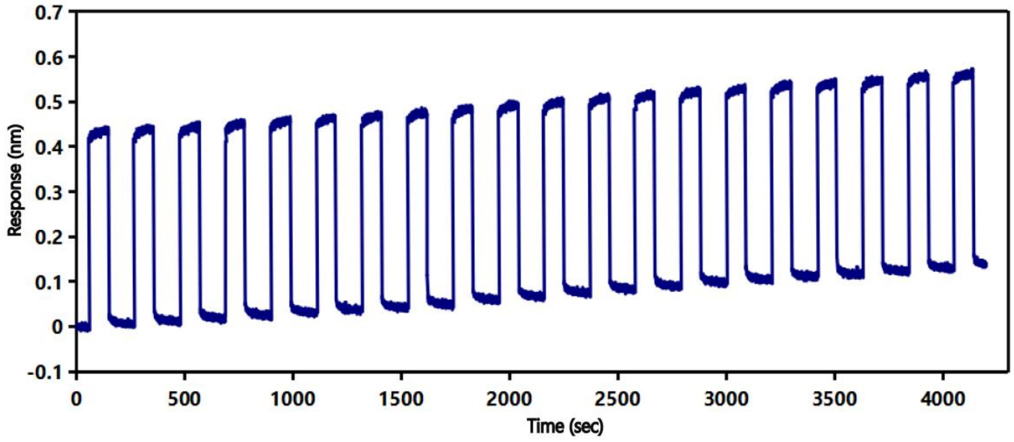

Figure S4. Measurement of the reusability of the aptasensor.

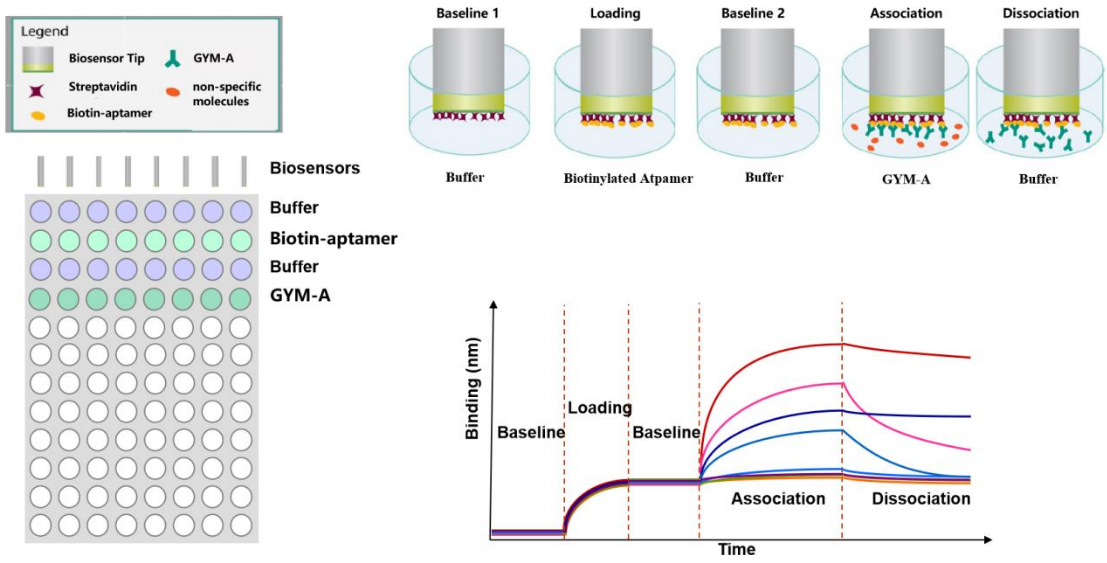

Figure S5. Schematic illustration of working flow of BLI-based aptasensor detecting GYM-A. BLI assays contains five steps: baseline 1 (60 s), loading (180 s), baseline 2 (60 s), association (90 s), and dissociation (60 s).
